# Interferon-γ Regulates the Death of *M. tuberculosis*-Infected Macrophages

**DOI:** 10.4137/jcd.s2822

**Published:** 2010-03-03

**Authors:** Jinhee Lee, Hardy Kornfeld

**Affiliations:** Department of Medicine, University of Massachusetts Medical School, Worcester, MA 01655.

**Keywords:** IFN-γ, *Mycobacterium tuberculosis*, macrophage, apoptosis, necrosis, HMGB1

## Abstract

We previously described a caspase-independent death induced in macrophages by a high intracellular burden of *Mycobacterium tuberculosis* (*Mtb*). This death, with features of apoptosis and necrosis, releases viable bacilli for spreading infection. Interferon (IFN)-γ promotes survival of macrophages with a low intracellular *Mtb* load by inhibiting bacterial replication. Macrophages in naïve hosts are unable to restrict *Mtb* replication following aerosol transmission, but IFN-γ is increasingly present when adaptive immunity is expressed in the lungs ∼2 weeks post-infection. We therefore investigated the effects of IFN-γ on macrophages challenged with *Mtb* at high multiplicity of infection (MOI). In contrast to the response at low MOI, IFN-γ accelerated the death of heavily infected macrophages and altered the characteristics of the dying cells. IFN-γ increased caspase-dependent DNA cleavage and apoptotic vesicle formation, but it also increased mitochondrial injury and release of LDH and HMGB1 in a caspase-independent manner. Adaptive immunity in tuberculosis (TB), mediated primarily by IFN-γ, has differential effects on *Mtb*-induced macrophage cell death depending on the intracellular bacillary load. While IFN-γ generally promotes host defense, our data suggest that its effects on heavily infected macrophages could also accelerate necrosis and spreading infection in TB disease.

## Introduction

The facultative intracellular pathogen *Mtb* is unable to grow in the extracellular spaces of the lung in newly infected hosts. Following inhalation by a naïve host, bacilli engulfed by alveolar macrophages suppress endosomal acidification and fusion with lysosomes that typically follows phagocytosis of most other microbes. The bacilli reside in a compartment with features of an early endosome where they have access to nutrients and proliferate, largely protected from host immunity. A growing body of evidence indicates that the death mode of infected macrophages is a critical event that can dictate the outcome of TB disease (reviewed in).^1^ Apoptosis benefits the host by removing the niche for bacillary replication,^2^ packaging *Mtb* antigens in apoptotic vesicles that enhance antigen presentation and cross-presentation by dendritic cells,^3^ and by killing intracellular bacilli.^4^,^5^ In contrast, macrophage necrosis is associated with unrestricted bacterial replication and severe disease.^6^

Macrophages harboring a low number of attenuated *Mtb* H37Ra or *M. bovis* BCG (≤10 bacilli per macrophage) become primed for tumor necrosis factor (TNF)-α death signals and classical caspase-mediated apoptosis.^4^,^7^ Virulent *Mtb* strains suppress this apoptosis response and grow inside the host cell until reaching a cytotoxic threshold (>20 bacilli per macrophage) at which point they trigger a non-classical death mode independent of TNF-α and caspases.^8^ The closely related live attenuated vaccine strain BCG is similarly cytotoxic when if introduced at high MOI. This death has some early features of apoptosis including nuclear pyknosis and plasma membrane phosphatidylserine (PS) translocation but with minimal DNA cleavage, no nuclear fragmentation and no apoptotic vesicle formation. Dying cells progress to necrosis within hours and release replication-competent bacilli to the extracellular space. This cell death mode constitutes an exit mechanism for *Mtb*, releasing bacilli for subsequent rounds of infection and intracellular replication and enabling the shift from intracellular to extracellular persistence that characterizes advanced TB disease.^1^

Host defense in TB relies on adaptive immunity and particularly on a broad range of activities stimulated in macrophages and dendritic cells by IFN-γ. The critical role of IFN-γ in TB immunity is well supported in animal studies^9^,^10^ and the susceptibility of humans with defective IFN-γ signaling.^11^–^13^ Among numerous defense-related functions, IFN-γ activates macrophages to kill or at least limit the growth of intracellular bacilli by inducing nitric oxide sythase and NADPH oxidase, and by accelerating phagolysosome fusion.^14^,^15^ The capacity of IFN-γ to restrict *Mtb* replication promotes macrophage survival after low MOI challenge by preventing bacilli from multiplying to the cytotoxic threshold,^16^ but in different contexts of cell death IFN-γ has also been shown to promote apoptosis. We therefore investigated the effect of IFN-γ activation on the fate of macrophages heavily infected with *Mtb*. In this physiologically relevant setting we found that IFN-γ increases macrophage cell death rather than survival. It induces caspase-dependent DNA cleavage and apoptotic vesicle formation but in a caspase-independent manner it also accelerates necrosis of infected cells as evidenced by release of lactate dehydrogenase (LDH) and high-mobility group box 1 protein (HMGB1).

## Materials and Methods

### Bone marrow-derived macrophage cultures

Bone marrow derived macrophages (BMDM) were generated from wildtype (WT) mice, TNF-α knockout (KO) mice, interferon regulatory factor (IRF)-1 KO mice, and signal transducer and activator of transcription (Stat1) KO mice all on the C57/BL6 background. Mice were purchased from Jackson Laboratory (Bar Harbor, Maine) except for TNF-α KO mice which were generously provided by Dr. L. Selin (University of Massachusetts Medical School, Worcester, MA). The use of animals for these experiments was reviewed and approved by the University of Massachusetts Institutional Animal Care and Use Committee. BMDM were prepared by culturing bone marrow cells in DMEM medium (Gibco, Gaithersburg, MD) supplemented with 10% L929 conditioned medium, 10% FBS (BioWhittaker, Walkersville, MD), 100 units/ml of penicillin, 100 mg/ml of streptomycin, and 2 mM glutamine (complete DMEM) for 7 days.

### Bacterial infections

Wild type *Mtb* Erdman, *Mtb* H37Rv and *M. bovis* BCG (Pasteur strain) were purchased from ATCC (Manassas, VA). A high intracellular burden of either strain induces the same cytotoxic response in macrophages.^8^ BCG was used for experiments more conveniently conducted with BSL-2 containment. The inclusion of studies with *Mtb* Erdman ensured that observations were not unique to only one strain of the *Mtb* complex, which includes *M. bovis*. Bacteria were grown at 37 °C with continuous agitation in Middlebrook 7H9 broth supplemented with 10% OADC, 0.2% glycerol and 0.05% Tween 80 (Fisher Scientific, Pittsburgh, PA). Bacteria in log growth phase were washed twice and resuspended with PBS containing 0.05% Tween 80. Bacterial concentration was determined by plating on 7H11 agar with OADC and counting colony forming units (CFU). Before infection, 1 ml of bacterial stock was thawed, dispersed using a cup sonicator (Branson Ultrasonics Corp., Danbury, CT) at a full power for 1 min, and then left for 20 min to permit clumps to settle. BMDM were plated in 8 well Lab-Tek tissue culture chamber slides (Nalge Nunc International, Naperville, IL) at 2 × 10^5^ cells per well for DAPI and TUNEL staining, or in 24 well plates at 5 × 10^5^ cells per well for mitochondrial staining and LDH release assay. Cells pretreated with IFN-γ (100 units/ml) (R&D systems, Minneapolis, MN) overnight were infected with *M. bovis* BCG or *Mtb* Erdman at MOI from 5–50 in antibiotic-free complete DMEM (37 °C, 3 hr). The concentration of IFN-γ used for these experiments was based on reported IFN-γ concentrations in the lungs of *Mtb*-infected mice^37^ converted to the specific activity of the recombinant cytokine, which corresponded to in vivo levels of 100 to 400 units/ml. IFN-γ was removed by washing two times with antibiotic-free complete DMEM before infection. In some experiments Z-VAD-fmk (R&D systems) was added at 10 μM to cell cultures 1 h prior to infection and maintained throughout the infection. After 3 h of infection, cells were then washed to remove unbound bacteria and further incubated for the indicated times in antibiotic-free complete DMEM.

### DAPI staining

Cells were washed twice with PBS and fixed with 4% paraformaldehyde (20 min, room temperature). Cells were next washed once with distilled water and stained with 4′,6-diamidino-2-phenylindole dihydrochloride (DAPI; Invitrogen, Carlsbad, CA) 1 μg/ml for 2 min. Cells were washed twice with distilled water and the slides were dried and mounted with Antifade Solution (Invitrogen) for fluorescence microscopy.

### Scanning electron microscopy

Following infection, cells were washed with PBS and fixed with 4% paraformaldehyde. Samples were processed at the Microscopy Imaging Lab, University of Massachusetts Medical School. Briefly, cells were washed with PBS and fixed with 4% paraformaldehyde (v/v)/2.5% glutaraldehyde (v/v) in 0.5 M Na phosphate buffer (pH 7.2) overnight at 4 °C. The next day the fixed samples were washed three times in fresh 0.5 M Na phosphate buffer (pH 7.0). The entire bottom of the well plates were cut out and dehydrated through a graded series of ethanol to 2 changes of 100% ethanol and critical point dried in liquid CO_2_. The dish bottoms with the dried cells were mounted onto aluminum stubs with silver conductive paste and sputter coated with gold/palladium (4 nm). The specimens were then examined using an FEI Quanta 200 FEG scanning electron microscope.

### ELISA and TUNEL

Apoptosis was measured by Cell Death ELISA assay kit (Roche, Indianapolis, IN) and terminal deoxynucleotidyl transferase (TdT)-mediated dUTP nick end labeling (TUNEL) (Roche). Differentiated bone marrow derived macrophages were detached from 100 mm petri dishes, counted, and plated in 8 well Lab-Tek tissue culture chamber slides (Nalge Nunc International, Naperville, IL) at 2 × 10^5^ cells per well. For Cell Death ELISA, culture medium was aspirated and cell treated with lysis buffer overnight at 4 °C. Antigen capture ELISA for histone/DNA complexes was performed according the manufacturer’s protocol. Results are expressed as fold differences in absorbance measured at 405 nm between experimental and control cultures. For TUNEL staining, cells were washed with PBS and fixed with 4% paraformaldehyde for 1 h. Fixed cells were permeabilized, incubated with TdT and fluorescein-labeled dUTP, and examined under the fluorescence microscope. Antigen capture ELISA to measure HMGB1 was kindly performed by J. Tian (Medimmune Inc., Gaithersburg, MD).

### Mycobacterial viability

BMDM were cultured in 24-well plates (2 × 10^5^ cells per well) and infected with *Mtb* Erdman (MOI 25). Cells were washed to remove unbound bacilli and further incubated in antibiotic-free media. Mycobacterial viability was measured 24 h post infection by lysing the cells in 0.25% saponin dissolved in PBS with 0.05% Tween 80 (20 min, 37 °C) to release intracellular bacilli. Cell culture supernatant was also collected to include mycobacteria released into media during the infection. Serial 10-fold dilutions of infected cell lysates were prepared in PBS with 0.05% Tween 80, and then 100 μl of each dilution was plated in duplicate on Middlebrook 7H11 agar plates. Colonies were counted 13 days and 21 days after plating.

### Mitochondrial membrane potential

After infection with *Mtb*, cells were incubated with 100 nM of MitoTracker CMXRos (Invitrogen) in complete media (37 °C, 20 min) and then washed twice with complete media. Cells were detached with 0.05% trypsin and 0.02% EDTA for 25 min, washed, and fixed with 1% paraformaldehyde. Flow cytometry was performed on an LSRII flow cytometer (BD Bioscience Pharmingen), 50,000 leukocyte-gated events were collected and data analyzed with FlowJo PC software (TreeStar, Inc.) Ashland, OR. Results are plotted as MitoTracker fluorescence vs. % of Max, which normalizes the number of events collected in overlaid samples. The % of Max is the number of cells in each bin divided by the number of cells in the bin that contains the largest number of cells.

### Measurement of LDH release

Infection-induced macrophage necrosis was determined by measuring LDH release using a commercial assay (Roche). BMDM infected with BCG for 3 h were washed and incubated in Opti-MEM (Invitrogen) containing 0.5% FBS for 6 h. LDH assay was performed according to a supplied protocol that includes culture supernatant from experimental and control wells, media control, and total cell lysates from wells incubated with 1% Triton X-100. Supernatant and reaction buffer were mixed for 10 min and then absorbance was measured at 490 nm. Data are expressed as % LDH release = (LDH activity in culture supernatant—LDH activity in media)/(LDH activity in total lysates—LDH activity in media) × 100%.

### Statistics

Data were analyzed by student’s *t*-test or one-way ANOVA followed by the Dunnett’s or Bonferroni’s multiple comparison post test, or two-way ANOVA using GraphPad Prism 5 (GraphPad, La Jolla, CA). Values of p < 0.05 were considered statistically significant.

## Results

### IFN-γ promotes DNA cleavage and apoptotic vesicle formation in heavily infected macrophages

**Figure 1:**
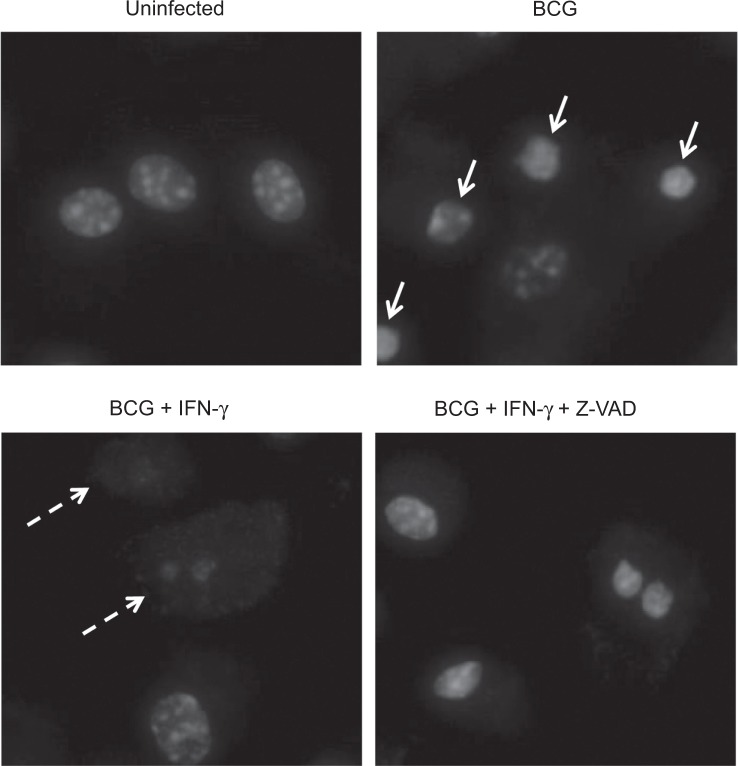
IFN-γ promotes the loss of chromosomal DNA. BMDM were pretreated with IFN-γ (100 units/ml) overnight and infected with BCG at MOI of 50 for 6 h. Cells were then fixed and stained with DAPI for microscopy (×400). Uninfected cells (*Top left*) show normal nuclear morphology. BCG-infected cells (*Top right*) show nuclear pyknosis (*Arrows*) typical of high-MOI cell death. Cells treated with IFN-γ prior to BCG infection (*Bottom left*) show near total loss of nuclear DAPI staining (*Dashed arrows*). The addition of Z-VAD-fmk 1 h prior to infection restored the nuclear morphology of BCG-infected cells treated with IFN-γ to that of cells infected with BCG in the absence of IFN-γ (*Bottom right*).

We investigated the effect of exogenous IFN-γ in the context of macrophage cell death induced by intracellular mycobacteria (MOI 25 or 50), expecting that the cytokine would enhance cell survival. In pilot studies, IFN-γ pretreatment appeared to rescue BMDM since it dramatically reduced propidium iodide staining of heavily infected cells identified by fluorescence microscopy (data not shown). However, DAPI staining revealed that rather than promoting survival, IFN-γ caused increased DNA cleavage of such magnitude that PI staining became unapparent. Nuclei of IFN-γ-treated BMDM were smaller and had dimmer DAPI staining after infection with BCG at MOI 50 than similarly infected cells without IFN-γ stimulation (Fig. 1). This effect of IFN-γ was caspase-dependent, since co-treatment with the pan-caspase inhibitor Z-VAD-fmk restored nuclear morphology to that of heavily infected cells not treated with IFN-γ (condensed but DAPI-bright). Consistent with the previous report that IFN-γ promotes survival of macrophages with a low intracellular bacillary burden,^16^ we saw no increase in apoptosis of IFN-γ pretreated macrophages challenged at MOI 5 (data not shown).

**Figure 2:**
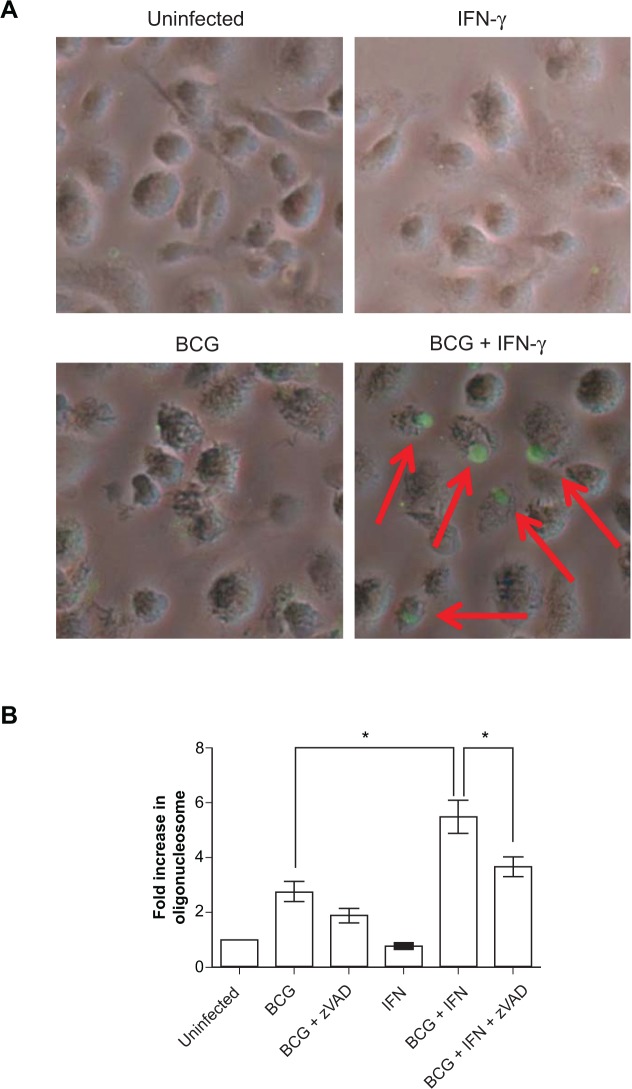
IFN-γ pretreatment increases chromosomal DNA fragmentation in BCG-infected cells. **A**) Cells were pretreated with IFN-γ overnight and infected with BCG at MOI of 50 for 6 h. Cells were then fixed and DNA strand breaks marked by fluorescent TUNEL. Phase contrast and fluorescence images were merged (x200). Red arrows indicate TUNEL positive cells. **B**) BMDM were pretreated with IFN-γ overnight and infected with BCG at MOI of 50 for 9 h. Z-VAD-fmk (10 μM) was added 1 h prior to infection. Data are expressed as mean fold increase in oligonucleosomes ± SEM of 4 independent experiments. *p < 0.05.

**Figure 3:**
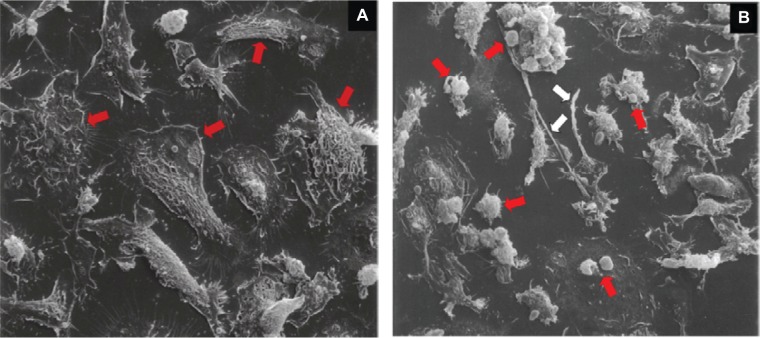
IFN-γ pretreatment increases membrane blebbing in *Mtb*-infected cells. BMDM were pretreated with IFN-γ overnight and infected with *Mtb* Erdman at MOI of 25 for 9 h. Cells were then fixed and prepared for scanning electron microscopoy (×500). **A**) *Mtb*-infected macrophages not treated with IFN-γ contained numerous necrotic cells (*Arrows*) with little evidence of apoptotic vesicle formation. **B**) Macrophages pretreated with IFN-γ had membranes blebs and apoptotic vesicles (*Red arrows*) as well as the “string phenotype” of apoptotic macrophages (*White arrows*) described by Winau et al.^38^

To corroborate the observations made with DAPI, we performed TUNEL staining of BMDM with similar conditions of BCG infection with and without IFN-γ stimulation. In the absence of IFN-γ pretreatment TUNEL staining was undetectable 6 h after challenge with BCG (MOI 50), confirming our previous observation that DNA cleavage does not accompany the nuclear pyknosis characteristic of high-MOI cell death (Fig. 2A). In contrast, TUNEL staining was observed in BCG-infected cells stimulated with IFN-γ (but not in uninfected cells similarly stimulated with IFN-γ). The positive TUNEL signals in cells pretreated with IFN-γ and infected with BCG were confined to nuclei rather than dispersed throughout cytosol, a pattern typical of apoptosis. Chromosomal DNA fragmentation was quantitatively measured by antigen capture ELISA of histone-complexed DNA fragments (mono- and oligonucleosomes). Consistent with the DAPI and TUNEL results, IFN-γ enriched the generation of nucleosomal particles in BCG-infected cells (Fig. 2B). The response to IFN-γ was blocked by Z-VAD-fmk, indicating that IFN-γ stimulated caspase-dependent DNase activity. In the absence of IFN-γ, high-MOI cell death has limited features of apoptosis that are clearly caspase-independent.^8^ The data presented here suggest that macrophages undergoing this atypical death process are primed to respond to IFN-γ with a shift towards features characteristic of caspase-mediated apoptosis. This was further supported by evidence that IFN-γ increased apoptotic vesicle formation, another hallmark of apoptosis that is generally absent in high-MOI cell death (Fig. 3). Despite the appearance of these apoptotic features, IFN-γ did not promote nuclear fragmentation in heavily infected macrophages (Fig. 1), indicating selectivity in the pro-apoptotic activity of this pleiotropic cytokine.

### IFN-γ stimulated DNA cleavage is dependent on Stat1 but independent of TNF-α or IRF-1

**Figure 4:**
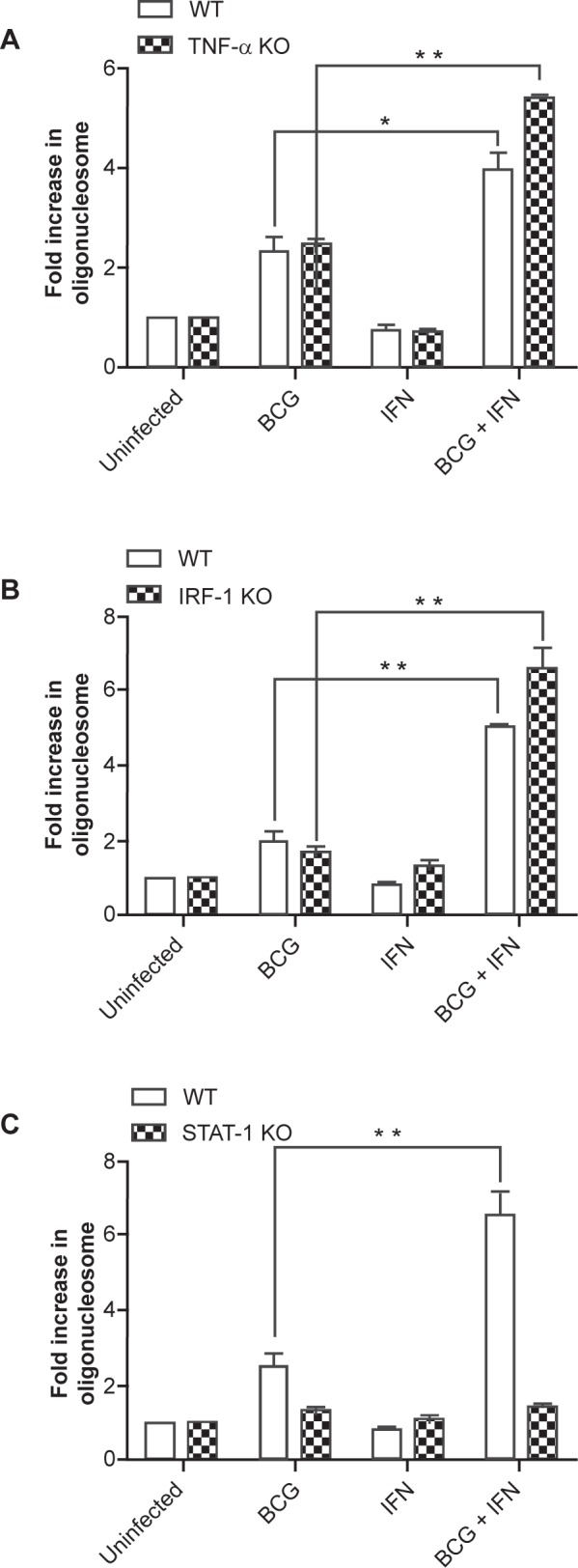
Chromosomal DNA cleavage stimulated in heavily infected macrophages by IFN-γ requires Stat1 but is independent of TNF-α or IRF-1. BMDM from TNF-α KO **A**) IRF-1 KO **B**) Stat1 KO **C**) and wild type (WT) mice were pretreated with IFN-γ overnight and infected with BCG at MOI of 25 for 9 h. Cells were then lysed and oligonucleosomes quantified by antigen-capture ELISA. Data are expressed as mean fold increase in oligonucleosomes ± SEM of three independent experiments. *p < 0.05; **p < 0.01.

The mechanism of macrophage cell death triggered by a high intracellular burden of *Mtb* complex bacilli is independent of TNF-α.^8^ Evidence that IFN-γ induces TNF receptor expression on tumor cells lines^17^ led us to speculate that IFN-γ might confer sensitivity to TNF-α in macrophage dying with a high intracellular bacillary load. However, macrophages from TNF-α KO mice were even more responsive than WT cells to IFN-γ-stimulated DNA cleavage after high-MOI challenge with BCG (Fig. 4A). This conclusively demonstrates that the pro-apoptotic effect of IFN-γ in these cells is independent of TNF-α.

The transcription factor IRF-1 mediates many of the downstream responses to IFN-γ signals, including apoptosis in some systems.^18^ To test whether the pro-apoptotic effect of IFN-γ in heavily infected macrophages is mediated by IRF-1, macrophages from WT and IRF-1 KO mice were compared. Similar to TNF-α KO mice, macrophages from IRF-1 KO mice were more responsive than WT cells to IFN-γ pretreatment (Fig. 4B), indicating that the pro-apoptotic effect of IFN-γ was independent of IRF-1. Of the more than 1,000 genes upregulated in macrophages by IFN-γ^19^ less than 50 may be independent of the IFN-receptor associated Stat1.^20^ Using macrophages from WT and Stat1 KO mice we confirmed that Stat1 is essential for the pro-apoptotic effect of IFN-γ in the context of high-MOI BCG-induced cell death (Fig. 4C).

### IFN-γ promotes necrosis of *Mtb*-infected macrophages

**Figure 5:**
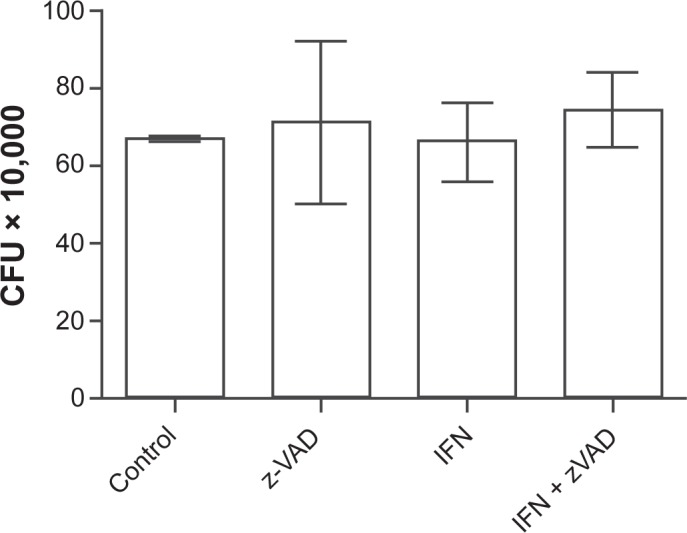
IFN-γ pretreatment does not affect the viability of intracellular bacteria. Cells were pretreated with IFN-γ overnight and infected with *Mtb* Erdman at MOI of 25 for 24 h. Z-VAD-fmk was added 1 h prior to infection. Data are presented as the mean CFU (×10,000) ± SD of three replicate wells.

Extrinsic, caspase-dependent apoptosis of infected macrophages reduces the viability of intracellular *Mtb*,^5^,^21^ while the caspase-independent death triggered by a high intracellular load of virulent *Mtb* has no such microbicidal effect.^8^ We therefore investigated the possibility that the pro-apoptotic effect of IFN-γ might be associated with concomitant antimicrobial activity. Untreated macrophages and macrophages pre-treated with IFN-γ, Z-VAD-fmk or both agents combined were infected with *Mtb* Erdman (MOI 25) and the number of bacterial colonies in each condition was measured 24 h post-infection. There was no difference in CFU between all four groups (Fig. 5), indicating that the apoptotic features of macrophage cell death induced by IFN-γ did not confer microbicidal activity in the dying cells.

**Figure 6:**
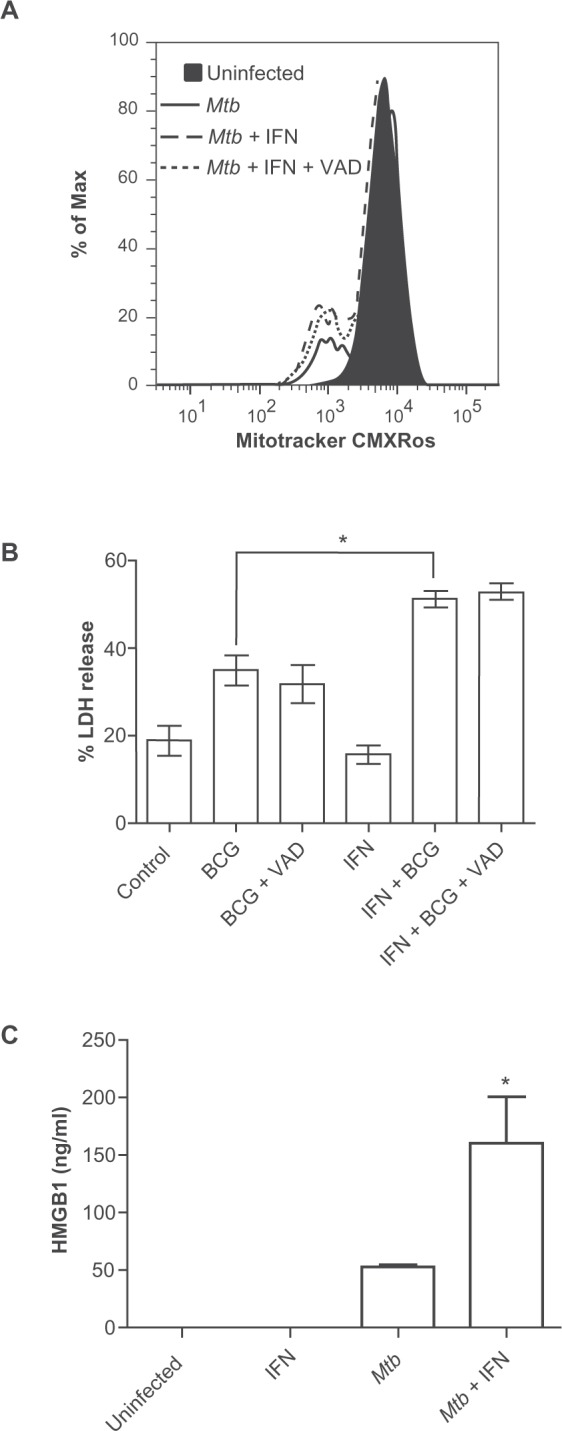
IFN-γ increases necrosis heavily infected macrophages as determined by loss of ΔΨ_m_ **A**) increased LDH release **B**) and increased HMGB1 release **C**) from heavily infected macrophages. **A**) Cells infected with *Mtb* Erdman at MOI of 25 for 3 h were stained with MitoTracker CMXRos to measure ΔΨ_m_. The filled peak corresponds to uninfected cells, the solid line corresponds to *Mtb*-infected cells, while the dashed and dotted lines correspond to *Mtb*-infected cells treated with IFN-γ or IFN-γ plus Z-VAD-fmk, respectively. Histograms are representative of three independent experiments. **B**) For LDH assays IFN-γ pretreated BMDM were infected with BCG at MOI of 25 for 9 h and then supernatant was collected for measurement of LDH. Data are means ± SEM of four independent experiments. **C**) For HMGB1 assays cells were infected with *Mtb* Erdman at MOI of 25 for 6 h. Culture supernatants were collected and HMGB1 was measured by antigen-capture ELISA. Data are presented as the mean ± SD from two independent experiments. *p < 0.05.

The macrophage cell death mode triggered by a high intracellular burden of *Mtb* complex bacilli has some early characteristics of apoptosis even in the absence of IFN-γ, but progresses to necrosis within hours. We speculated that the failure of IFN-γ to promote killing of *Mtb* in our experiments might be due to rapid necrosis of the heavily infected cells, disrupting the compartments responsible for intracellular antimicrobial activity. Mitochondria play a central role in many cell death modes and the loss of mitochondrial inner transmembrane potential (ΔΨ_m_) is associated with necrosis.^22^ In heavily infected macrophages, IFN-γ increased this dissipation of ΔΨ_m_ as measured by cationic dye release and this effect of IFN-γ was not inhibited by Z-VAD-fmk (Fig. 6A). In macrophages heavily infected with pathogenic mycobacteria, IFN-γ exerts a caspase-independent effect to accelerate mitochondrial injury linked to necrosis. To confirm that the dissipation of ΔΨ_m_ induced by IFN-γ resulted in increased cellular necrosis we measured LDH release from of heavily infected BMDM (Fig. 6B). Infection with BCG increased LDH release compared to uninfected cells and this effect of BCG was no different in the presence or absence of Z-VAD-fmk. Consistent with the mitochondrial injury data, LDH release from infected cells was further increased by pretreatment with IFN-γ and this pro-necrotic effect of IFN-γ was not inhibited by Z-VAD-fmk. Thus, IFN-γ has dual effects in heavily infected macrophages; it stimulates caspase-mediated apoptotic activities (DNA cleavage and apoptotic vesicle formation) but simultaneously accelerates necrosis (dissipation of ΔΨ_m_ and LDH release).

HMGB1 is a non-histone nuclear structural protein that may be preferentially released from dying cells in the setting of necrosis but not apoptosis,^23^,^24^ although this distinction has become less certain with ongoing investigations.^25^ We measured the levels of extracellular HMGB1 in cultures of BMDM infected with *Mtb* Erdman (MOI 25) in the presence or absence of IFN-γ. HMGB1 was not detectable in supernatant of uninfected BMDM or uninfected cells treated with IFN-γ but it was released from cells heavily infected with *Mtb* (Fig. 6C). Interestingly, IFN-γ pretreatment more than doubled the amount of HMGB1 released from infected macrophages. This result was consistent with the dramatic impact of IFN-γ on nuclear morphology (Fig. 1).

## Discussion

Host-pathogen interactions in TB resulting in death of infected macrophages or the infecting microbes are central to disease pathogenesis and complex in nature. We report a novel effect of INF-γ to regulate the death mode of macrophages heavily infected with *Mtb* complex bacilli including *M. bovis* BCG and *Mtb* Erdman. In the absence of INF-γ, a high intracellular bacillary load triggers a lethal chain of events that include nuclear pyknosis and PS externalization commonly seen in apoptosis but with rapid progression to necrosis. This death mode, which is caspase-independent and possibly a result of lysosomal injury, is postulated to serve as an exit mechanism for *Mtb* to facilitate spreading infection.^8^ In vitro studies of *Mtb*-induced macrophage cell death have mostly been conducted in the absence of exogenous cytokine signals. In the present study we found that INF-γ (a cytokine present in high levels in lungs in established TB disease) stimulates caspase-dependent chromosomal DNA fragmentation and apoptotic vesicle formation in heavily infected macrophages, but it also increases mitochondrial injury and LDH release indicative of necrosis and it increased the release of HMGB1. These results indicate that adaptive immunity, through IFN-γ, can alter the features of infection-induced cell death in TB. Whether this outcome predominantly benefits the host or the pathogen in TB remains to be determined.

Apoptosis is considered a defense against intracellular pathogens and it has been established that virulent *Mtb* strains suppress the macrophage apoptotic response mediated by TNF-α and caspases. Mutant *Mtb* strains unable to block apoptosis have reduced virulence in vivo^26^ and efficiently promote adaptive immunity presumably through efferocytosis of antigen-loaded apoptotic vesicles by dendritic cells.^3^ However, some consequences of apoptosis could benefit the pathogen for example by usurping efferocytosis for dissemination to naïve phagocytes.^27^ Necrosis of *Mtb*-infected macrophages appears to be an adverse outcome for the host. Necrosis is postulated to release infecting bacilli from the confinement of apoptotic membranes, to recruit naïve phagocytes for spreading infection and to promote the tissue-damaging inflammation that is characteristic of advanced pulmonary TB and a prerequisite for aerosol transmission to a new host. IFN-γ stimulates a caspase-dependent apoptotic response in heavily infected macrophages that could potentially mediate direct antimicrobial activity and package *Mtb* antigens in apoptotic vesicles to the benefit of the host. However, it also accelerates ΔΨ_m_ dissipation and necrosis, suggesting that *Mtb* might limit any protective effect of IFN-γ on the macrophage death mode and possibly exploit the attendant increased necrosis.

Our studies revealed that IFN-γ increases HMGB1 from *Mtb*-infected macrophages. HMGB1, a non-histone nuclear protein that modulates the transcription factor interactions with chromosomal DNA,^23^ also functions as an extracellular cytokine released passively from dying cells or by a regulated process of secretion. HMGB1 binding to nucleosomes is strengthened during apoptosis, leading to the early postulate that its release is a biomarker of necrosis. However, the exclusive association of HMGB1 release with necrosis has been challenged.^25^ HMGB1 release in the context of mycobacterial infection has been reported,^28^ although the mechanism of release (passive or active secretion) was not described. HMGB1 release in our experiments was linked to cell death as we detected the protein in culture supernatant 6 h post-infection but not uninfected cells, and regulated secretion from macrophages was reported to require at least 16 h.^24^ As a death-associated molecular pattern, HMGB1 has been linked to immunogenic cell death^29^ but also to immune tolerance induced by apoptotic cells, possibly based on differential activities of oxidized and reduced forms of the protein.^30^ Differential release of HMGB1 in TB lesions would be expected to influence the composition and function of inflammatory cells recruited to sites of infection. Since IFN-γ levels rise only after priming of adaptive immunity to *Mtb* its impact on HMGB1 release would most likely increase the immunopathology of pulmonary TB.

The effects of IFN-γ on the survival or death of *Mtb*-infected macrophages are dramatically different depending on the number of intracellular bacilli. IFN-γ activates a range of antimicrobial functions that suppress *Mtb* replication after low-MOI challenge. By blocking bacterial proliferation to a lethal intracellular bacillary load, IFN-γ acts as a survival factor for macrophages that have engulfed fewer than 10 bacilli.^16^ Our data presented here show that macrophages with a high intracellular bacillary load become primed to respond to Stat1-mediated IFN-γ signals with manifestations of apoptosis and necrosis. IFN-γ is known to induce a number of proapoptotic genes including IRF-1, the death associated proteins, protein kinase R, cathepsin B, D, and L, Fas/Fas ligand, TNF-α receptor, TRAIL, Bak and caspases 1, 3, 4, 7, 8 and 10.^31^,^32^ One or more of these genes might be responsible for the pro-apoptotic and/or pro-necrotic effects of IFN-γ revealed in our experiments. The lack of dependence on IRF-1 we observed could eliminate some of these candidates such as caspase 1 (which has been implicated in several reports of IFN-γ-induced apoptosis)^33^–^35^ and caspase 8.^36^

In summary, our study has revealed IRF-1 independent pro-apoptotic and pro-necrotic activities of IFN-γ functioning specifically in the context of macrophages heavily infected with *Mtb* complex bacilli. These biological effects were observed with levels of INF-γ comparable to those we have measured in lungs of *Mtb*-infected mice in vivo.^37^ Our findings highlight the complex regulation of cell fate in TB and suggest that multiple death pathways may operate in parallel within infected macrophages. Adaptive immunity is essential to control TB disease and IFN-γ production by CD4^+^ T cells is the most critical element of a host defense that enforces lifelong latency for ∼90% of *Mtb*-infected individuals. The bacillus has evolved to persist in the host despite robust T_H_1 biased cell-mediated immunity and to cause active TB disease in ∼10% of infected individuals. In the equilibrium between host and pathogen, protective factors such as IFN-γ may be subverted to promote disease under particular conditions.
